# Impact of Early Season Jump Loads on Neuromuscular Performance in Division I Volleyball: Analyzing Force, Velocity, and Power From Countermovement Jump Tests

**DOI:** 10.1155/tsm2/7216781

**Published:** 2025-04-04

**Authors:** Gabriel J. Sanders, Stacie Skodinski, Corey A. Peacock

**Affiliations:** ^1^Department of Exercise Science, University of Cincinnati, Cincinnati, Ohio, USA; ^2^Department of Strength and Conditioning, University of Cincinnati, Cincinnati, Ohio, USA; ^3^Department of Kinesiology, Nova Southeastern University, Fort Lauderdale, Florida, USA

**Keywords:** alarm phase, force plate, jumping

## Abstract

The study investigated daily jump load variations on neuromuscular fatigue in nine NCAA Division I female volleyball athletes during the first 22 days of the season. Using force plates and inertial measurement units, data from 17 sessions were analyzed to assess relationships between jump loads and neuromuscular performance. Pearson's correlations were calculated to assess the relationships between force, velocity, and power force plate metrics and jump variables (duration in minutes, total jump counts, and jump counts greater than 38.1 cm (Jumps 38+) and 50.8 cm (Jumps 50+)). Nine out of 14 force metrics showed weak-to-moderate negative correlations with Jumps 50+, indicating as the highest intensity of jump counts increased and force production decreased (*r* ranges from −0.194 to −0.570; *p* ≤ 0.025 for all). In contrast, nine out of 16 velocity and power metrics showed weak-to-moderate positive correlations with Jumps 50+ (*r* ranges from 0.175 to 0.466; *p* ≤ 0.044 for all). In total, 29 out of 36 force plate metrics were significantly correlated to Jumps 50+, the highest intensity jump threshold assessed. Monitoring high-intensity jump loads provides a more accurate and nuanced assessment of neuromuscular performance and fatigue than total jump counts or session duration, with implications for optimizing athlete readiness and performance.

## 1. Introduction

Monitoring jump loads in elite volleyball is commonplace for optimizing performance and minimizing injury risk [1–6]. Metrics such as total jump counts and high-intensity jump counts are valuable indicators of an athlete's workload, providing insight into their physical demands during both training and competition [2, 3, 6–9]. Notably, the number of high-intensity jumps in practice can be two to three times greater than in games, raising concerns about the impact of the increased load, especially early in the season, on neuromuscular fatigue [8]. Excessive high-intensity jumps are known to increase fatigue and reduce performance, and consistent jump loads, regardless of intensity, have been linked to increased fatigue as shown by declines in peak power and jump height in countermovement jump (CMJ) tests [8, 10–12]. Additionally, cumulative training stress over the season has been found to impair neuromuscular function and elevate injury risk in uninjured Division I female athletes, suggesting that the initial stress of the season could have lasting physiological effects [2, 3, 13]. Despite these findings, it remains uncertain how different intensities of jump activity impact daily neuromuscular fatigue and performance throughout the initial phase of the preseason and the season.

The increasing use of wearable technology, such as accelerometers and inertial measurement units (IMUs), has added convenience to monitoring jump performance [6]. These devices are portable and easy to use in real-world settings, making them popular for tracking athlete performance. However, wearable tech comes with challenges, including potential data variability due to sensor placement, athlete movement, and environmental conditions [14]. Unlike force plates, which provide highly accurate measurements of force production and timing, wearable devices can sometimes lack the precision needed to detect subtle changes in neuromuscular function [15, 16]. Additionally, inconsistencies in the algorithms used by different manufacturers to calculate jump metrics such as propulsion and braking force can lead to data discrepancies [17]. Therefore, while CMJ tests, especially those utilizing force plates, are valuable tools for monitoring neuromuscular fatigue, practitioners should exercise caution when relying solely on wearable technology or exclusively on CMJ tests. Instead, they should consider integrating both methods alongside other ideal approaches for tracking and analyzing data to ensure comprehensive and accurate assessments. The CMJ test is widely used in athlete monitoring, particularly for assessing neuromuscular status and detecting fatigue in sports such as volleyball [3, 11, 12, 18]. Its high practicality and low physiological strain allow for repeated assessments of multiple athletes within short time frames, making it an attractive option for ongoing performance monitoring. While the CMJ test can provide valuable insights into various components of neuromuscular function, the specific variables most sensitive to neuromuscular fatigue due to jump loads are still debated [18]. Further, from an analysis standpoint, averaging multiple jumps within a test session can improve the reliability of CMJ measurements, thereby increasing the test's sensitivity to detect fatigue [18–20].

The transition from the off-season to the beginning of the preseason and the competitive season in volleyball is a critical period that warrants thorough investigation due to the sudden and significant increase in jump loads [13]. Research indicates that injuries and fatigue are particularly prevalent at the beginning of the season, as athletes often transition from lower-intensity off-season training to the high-intensity demands of competitive play [21]. This abrupt increase in workload can overwhelm athletes who may arrive at the preseason under-conditioned or not fully prepared for the intense physical demands of the season [1]. Additional challenges can further stress athletes such as variable practice scheduling, athlete compliance with training and recovery recommendations, and variability in individual recovery [22]. By properly managing workloads and monitoring physical stress levels, especially at the start of the season, coaches and sports scientists can develop strategies that can enhance athlete resilience, reduce the risk of injury, and promote long-term health and performance [1]. Addressing these challenges and refining data collection methods can lead to more effective workload management practices, ultimately supporting athlete well-being throughout the entire season.

Although force plates are among the most widely used tools for assessing neuromuscular performance in sports-specific settings, there remains a scarcity of the scientific literature investigating changes in CMJ performance among female volleyball athletes in relation to daily jump loads. Although a longitudinal analysis in NCAA Division-I men's basketball has explored external workload data and force plate metrics, this study provides insights that address the specific research gap in volleyball. [23]. In particular, the neuromuscular demands associated with the daily high volumes of repeated jump characteristic of female volleyball athletes remain understudied. Further, the previous study did not focus on alterations in external workloads and CMJ performance at the onset of the season. Preseason training at the beginning of a season is often marked by an abrupt increase in physical demand, commonly referred to as the shock phase of training [2, 24, 25]. Anecdotal observations from practitioners suggest that many athletes return to campus and begin the preseason under-conditioned, which may heighten their risk of injury or negatively affect their performance. Therefore, the purpose of this study is to examine the relationship between daily jump loads and neuromuscular performance during the initial phase of a volleyball season. Specifically, the study aimed to assess how variations in jump counts at different jump intensities impact neuromuscular performance within the first month of a competitive season.

## 2. Materials and Methods

The study retrospectively evaluated data collected from nine NCAA Division I female volleyball athletes (*N* = 9, ages: 19.4 ± 1.3 years old, and height: 184.0 ± 7.1 cm) during the first 22 days of the competitive season. Weight was not included in the analysis due to university restraints in body composition data collection with athletes. All athletes that participated in the study were physically cleared by the team physician. The inclusion criteria required athletes to be free of injury, participate fully in each training session or game, and remain injury-free the following morning to complete two CMJs on a dual force plate system. Due to two mandatory days off, one abrupt schedule change, and three periods of time in which the CMJ test could not be performed the morning after a training session, a total of 17 days of data from daily jump loads and CMJ tests were included in the analysis, consisting of three matches and 14 practice sessions. Daily jump loads were measured using a wearable device, and corresponding CMJ tests were conducted the following morning. Participation was part of the strength and conditioning program at the university and part of normal collegiate varsity athletics participation. The study and analysis were approved by the university institutional review board.

### 2.1. Data Collection

Daily jump loads were monitored using the validated inertial measurement unit, a microsensor device equipped with a 3-axis accelerometer, gyroscope, and magnetometer (VERT 3, Fort Lauderdale, FL, USA) [5, 26, 27]. The device was securely positioned at the top of the iliac crest using a snug band, in accordance with the manufacturer's instructions. Since volleyball is played indoors, the wearable device, which did not include GPS-based metrics, was worn by each athlete during every practice and game throughout the season.

### 2.2. CMJ Testing

To assess neuromuscular fatigue, CMJ tests were conducted the first day of preseason training prior to any practice and then prior to practice every morning at approximately 8 a.m. using a validated, portable dual force plate system (Hawkin Dynamics, Westbrook, ME, USA), which operates at a sampling rate of 1000 Hz [28]. These tests were conducted in the morning prior to any organized warm-up or additional training session by the strength coach. A warm-up was not completed to align with evidence suggesting that lower-limb warm-ups can improve CMJ performance through neural mechanisms, independent of alterations in muscle activity, potentially masking signs of fatigue [29].

For each CMJ test, athletes were required to stand motionless on the force plates with their hands on their hips, allowing the system to measure their body weight in Newtons. Upon completion of this measurement, the system emitted a beep, signaling readiness for the jump. Athletes were then instructed to perform a maximal effort CMJ with their hands remaining on their hips. Each session consisted of two jumps, and the average of these two attempts was used for analysis. The force plate system recorded variables such as force, velocity, and power across the phases of the jump and the landing, which were later analyzed in relation to the daily jump load data. By aligning daily jump loads with the following morning's CMJ results, this study aimed to identify correlations between training load and neuromuscular performance, providing insights that could enhance training strategies and workload management during the critical early phase of the season.

### 2.3. Data Analysis

The data analysis plan involved a systematic process to merge and align the daily jump load data from the IMUs with the neuromuscular performance data collected from the dual force plate system. This alignment was crucial for investigating the relationship between the athletes' jump loads on one day and their CMJ tests recorded the following morning. The daily jump load data, including total jump counts and high-intensity jump counts, were recorded by the VERT devices worn by each athlete during practices and matches. These data were exported and organized on a daily basis. Simultaneously, the CMJ tests were conducted the next morning, with each athlete performing two maximal effort jumps on the dual force plate system. The force plate system captured detailed neuromuscular performance metrics such as propulsion, braking forces, and the rate of force development.

To ensure consistency and reliability, the two CMJ trials performed by each athlete were averaged to produce a single set of CMJ metrics for each testing session. This average was used to reduce variability and provide a more accurate representation of the athlete's neuromuscular performance.

Once the daily jump load data and the corresponding next-morning CMJ data were collected, they were merged using a unique identifier for each athlete. This process allowed the researchers to align the workload data from one day directly with the CMJ performance metrics from the following morning. By aligning these datasets in this manner, the study was able to explore time-lagged relationships between the previous day's jump loads and the subsequent morning's CMJ tests, providing insights into how jump load impacts neuromuscular function. There was a total of 134 observations of data collected from the 9 athletes across 17 training sessions and CMJ tests throughout the 22 days, in addition to the pre CMJ tests that were conducted prior to the first practice of the season. This approach allowed for a comprehensive analysis of the data, facilitating the investigation of correlations and potential relationships between jump loads and CMJ performance.

### 2.4. Statistical Analysis

Descriptive statistics were calculated for all jump and force plate variables collected during the first 22 days of the volleyball season. The dependent variables included total jump counts, high-intensity jump counts (Jumps 38+ for jumps > 38.1 cm and Jumps 50+ for jumps > 50.8 cm, categorized by the manufacturer), CMJ height, CMJ depth, as well as all force, velocity, and power metrics. Preseason force, velocity, and power force plate measures were reported separately to establish baseline CMJ performance levels for the athletes.

Prior to analysis, the normality of each variable was assessed using the Shapiro–Wilk test. This alignment allowed for the calculation of proper correlations between the workload from the previous day and the neuromuscular performance observed the next morning. Pearson correlation coefficients were used to assess the strength and direction of the relationships between jump loads and CMJ metrics. Three variables violated normality, relative to minimum displacement, average propulsion velocity, and peak breaking power. Furthermore, to compare the proportion of significant correlations (or nonsignificant) across different jump variables (duration, total jumps, and Jumps 38+ and Jumps 50+), Fisher's exact test was utilized, and odds ratios were calculated for each to determine which jump variables had significantly different proportions of significant correlations. Fisher's exact test is particularly suitable for data with small sample sizes and low frequencies in contingency tables, which is common in studies involving high-performance athletes where collecting large-scale data can be challenging [30]. Statistical significance was set a priori at *p* < 0.05. All statistical analyses were performed using IBM SPSS Statistics version 29.0.

## 3. Results

### 3.1. Baseline

Baseline measurements for force, velocity, and power, obtained from preseason force plate tests, were separately documented to establish initial CMJ performance levels for the athletes (see Table 1).

### 3.2. Force-Based Metrics

The relationships between jump variables and various force plate metrics revealed distinct patterns across the different jump variables, with Jumps 38+ and Jumps 50+ metrics showing stronger correlations, and Pearson's *r* and *p* values are provided in Table 2 for all jump and force metrics. The only significant correlation for duration was between peak relative braking force. There were no significant correlations between total jumps and any force plate measures.

Further, for Jumps 38+, negative correlations were observed with braking RFD, force at minimum displacement, and peak braking force. As a group, these findings indicate that as the jump counts of Jumps 38+ increase, there is a corresponding decrease in the magnitude of these braking-related force metrics, suggesting potential neuromuscular fatigue or adaptation. Additionally, Jumps 38+ showed a significant positive correlation with relative peak landing force, indicating that higher jumps may be associated with greater relative peak landing forces.

Interestingly, the strongest correlations were observed for Jumps 50+. Significant negative correlations were found with braking RFD, force at minimum displacement, peak and average braking force, and peak propulsion force. These results align with the findings for Jumps 38+ but suggest that a stronger relationship is more pronounced when greater jump counts occur at higher jump intensities.

### 3.3. Velocity-Based Metrics

The relationships between jump variables and velocity-based metrics and reactive strength metrics from the force plates indicated that Jumps 38+ and Jumps 50+ show weak-to-moderate correlations with velocity-based metrics, the reactive strength index (RSI), and the modified RSI (mRSI). Pearson's *r* and *p* values are provided in Table 3. For duration and total jumps, there were no significant correlations. Thus, duration and total jump counts do not influence the velocity or reactive strength and are less impactful on neuromuscular performance. In contrast, Jumps 38+ showed significant positive correlations, although weak, with takeoff velocity and peak velocity, indicating that as the jump count of Jumps 38+ increases, so does velocity.

Interestingly, the strongest correlations were observed for Jumps 50+. At the group level, significant positive correlations were found with takeoff velocity, peak velocity, and average propulsive velocity, indicating that higher jump counts at greater intensities are more strongly associated with faster velocities and greater reactive strength. Additionally, a significant negative correlation was observed with peak braking velocity, suggesting that as jump intensity increases, the peak braking velocity decreases, possibly reflecting neuromuscular adaptations to higher intensity jump loads.

### 3.4. Power-Based Metrics

Following the same pattern as force and velocity, the correlations between jump variables and power metrics from the force plates revealed Jumps 38+ and Jumps 50+ metrics showing stronger correlations. Pearson's *r* and *p* values are provided in Table 4 for all jump and power metrics.

There were no significant correlations between duration, total jumps, and any power metrics. In contrast, Jumps 38+ demonstrated several significant correlations with power metrics. Positive correlations were observed with jump height and flight time, while notable negative correlations were found with jump momentum, countermovement depth, and average propulsive power, indicating a decrease in momentum and propulsive power as the frequency of higher jumps increases. Meanwhile, there were low and moderate positive correlations for landing stiffness and stiffness, respectively.

The strongest correlations were observed for Jumps 50+. Significant positive correlations were identified with jump height, flight time, landing stiffness, stiffness, and peak relative propulsive power, suggesting that as the count of higher-intensity jumps increases, there is a corresponding increase in these metrics. Contradictory negative correlations were also noted with jump momentum and average propulsive power, indicating that higher jump counts at greater intensities may reduce these power metrics.

### 3.5. Fishers Exact Test

The results indicate a variable probability of observing significant correlations across different jump types relating to force plate measures (Table 5). Notably, jump counts at high intensities such as Jumps 38+ (odds ratio of 4.86; *p*=0.001) and Jumps 50+ (odds ratio of 12.17; *p*=0.001) are more frequently associated with significant correlations, whereas duration (odds ratio of 0.028; *p* < 0.001) and total jumps (odds ratio of 0.00; *p* < 0.001) are associated with nonsignificant correlations to force plate measures. These results underscore the need to consider the specific type of jump when analyzing the significance of correlations in sports performance data.

## 4. Discussion

This is the first study to examine jump loads at varying intensities and their relationship to CMJ performance the following morning during the preseason phase of the season. The primary findings revealed complex relationships between jump variables (such as duration, total jumps, Jumps 38+, and Jumps 50+) and different force, velocity, and power-based metrics measured via force plates. The most significant negative correlations were observed between force-based metrics and high-intensity jumps (Jumps 38+ and Jumps 50+), specifically braking RFD (*r* = −0.478), jump momentum (*r* = −0.220), and CMJ depth (*r* = −0.348). These negative correlations suggest that as the number of high-intensity jumps increases, force production decreases during subsequent CMJ tests the following morning, potentially indicating the onset of neuromuscular fatigue or adaptive responses to the increased demands.

Conversely, velocity-based metrics demonstrated primarily positive correlations with high-intensity jump counts, with Jumps 50+ being correlated with faster takeoff and peak velocities (*r* = 0.458; *p* ≤ 0.001 for each). The observed positive correlations between high-intensity jump counts and velocity-based metrics suggest that, at the group level, athletes with higher exposures to intense jumps tend to demonstrate greater speed and explosiveness in CMJ performance. Further, RSI and mRSI metrics were positively correlated with Jumps 50+, the highest intensity of jump counts, reinforcing the idea that jump intensity is a key driver of performance enhancements or perhaps these athletes who engage in a greater number of high-intensity jumps have the ability to maintain velocity, even if force production declines. Similarly, Cabarkapa et al. [11] found eccentric mean power, peak power, and peak velocity increased throughout the season, which supports the positive relationship in power metrics in the current study potentially reflecting beneficial neuromuscular adaptive responses by athletes to the progressively increasing demands of their training and competition schedules. However, the negative correlations with jump momentum and CMJ depth suggest a potential cost to these improvements, highlighting the complex interplay between different neuromuscular performance variables. These findings underscore the importance of focusing on monitoring the intensity of jumps combined with force-based metrics or CMJ depth and momentum rather than solely jump volume or duration when assessing neuromuscular performance and fatigue.

Fisher's exact test further highlighted the importance of jump intensity, showing that high-intensity jumps (Jumps 38+ and Jumps 50+) are more frequently associated with significant correlations, while duration and total jumps are less likely to yield meaningful associations with force plate measures after a day of practice, training, or a competition. It may be advisable for practitioners to focus on managing higher intensity jump counts to improve force production and maintain optimal neuromuscular readiness. Moreover, these findings underscore the complexity of the relationships between jump variables and neuromuscular performance and suggest that a combination of metrics, particularly those related to force and potentially countermovement depth and braking RFD, may be the most effective in identifying signs of neuromuscular fatigue.

Previous research suggests that force plate metrics, particularly measures such as braking RFD, peak propulsive force, and landing stiffness, are effective in identifying neuromuscular fatigue patterns [18, 19, 31]. These metrics are sensitive to changes in an athlete's neuromuscular status, as braking RFD and peak propulsive force could indicate fatigue after increased high-intensity jump counts, while increases in landing stiffness may reflect a compensatory mechanism to manage fatigue in rugby and Australian rules football [19, 31]. In the current study, similar patterns were observed, particularly with significant negative correlations between high-intensity jumps (Jumps 38+ and Jumps 50+) and braking RFD, as well as with peak braking force (*r* = −0.570) and peak propulsive force (*r* = −0.498), suggesting the onset of neuromuscular fatigue. The current study also showed significant positive correlation, albeit weak, between the highest intensity jumps and landing stiffness (*r* = 0.175), which may indicate an adaptive response or the previously postulated compensatory mechanism rather than fatigue. These findings align with previous research and highlight the complex and sometimes contradictory nature of neuromuscular fatigue indicators, emphasizing the need for a nuanced interpretation of force plate metrics.

Total jump counts were not ideal indicators of neuromuscular fatigue in volleyball because they fail to account for the intensity of the jumps, which is a critical factor in the onset of fatigue. In volleyball, different player positions engage in varying intensities of jumps, with front-row players such as middle blockers and outside hitters performing more high-intensity jumps compared to back-row players, who typically engage in less frequent and lower-intensity jumps [9]. This variation means that total jump counts alone do not provide a complete picture of an athlete's workload or their potential for neuromuscular fatigue. It is the intensity of the jumps, rather than just the quantity, that primarily contributes to fatigue, as high-intensity efforts are more taxing on the neuromuscular system [31]. Therefore, relying solely on total jump counts may overlook critical aspects of an athlete's workload, leading to an incomplete assessment of fatigue.

In volleyball, measuring duration in terms of minutes spent in practice, training, or game sessions is not an ideal indicator of neuromuscular fatigue because it does not account for the intensity of activity, which seems to be the primary driver of fatigue. High-intensity efforts, such as explosive jumps and sprints, impose significantly greater demands on the neuromuscular system than lower-intensity activities, regardless of the total duration [31, 32]. However, duration may still hold value when combined with a subjective metric such as the rating of perceived exertion (RPE) for volleyball athletes [33]. Session RPE (sRPE) is a commonly used metric by practitioners to estimate training load, as it is weighted by duration and perceived exertion, but it may not adequately reflect neuromuscular fatigue either [34]. The variability in how athletes perceive exertion might be more cardiovascular-related and not neuromuscular-related [35, 36], but previous research suggests that RPE has value in volleyball [37]. Thus, integrating both subjective measures (i.e., RPE) and objective measures (i.e., jump counts and force plate metrics) could offer a more comprehensive understanding of an athlete's fatigue status and internal training load.

While this study offers valuable insights into the relationships between high-intensity jump loads and neuromuscular performance in volleyball athletes, it is not without limitations. The wearable technology did not assess other relevant metrics such as change of direction, heart rate, speed of movements, etc. Further, sleep quantity and quality and nutrition may have provided valuable information combined with the jump loads and their relationship to neuromuscular function. Furthermore, the study's reliance on a small sample size from a single team during the first month of the season may affect the generalizability of the findings to the full season. Future research should aim to include additional external and internal metrics, expand the sample size, and assess these relationships throughout the season on a regular or semiregular basis. By addressing these gaps, future studies can contribute to more comprehensive workload management strategies.

## 5. Conclusion

Assessing neuromuscular performance and fatigue in volleyball athletes is complex; however, the results suggest that the intensity of jumps may negatively alter CMJ depth, momentum, and force-based metrics, while positively altering velocity and power-based metrics. Moreover, monitoring high-intensity jump counts offers more accurate insights to neuromuscular performance than simply counting total jumps or measuring session duration. Additional research is warranted to better understand the nuanced relationships between jump load and neuromuscular performance throughout a complete season.

## Figures and Tables

**Table 1:** Reports on all preseason force plate measures.

Force metrics	Mean ± SD
Braking RFD (N/s)	4502.8 ± 2361.1
Force at min displacement (N)	1544.5 ± 281.9
Relative force at min displacement (%)	210.7 ± 23.7
Average braking force (N)	1248.8 ± 231.0
Average relative braking force (%)	170.4 ± 19.7
Peak braking force (N)	1559.0 ± 290.5
Peak relative braking force (%)	212.6 ± 25.1
Average propulsive force (N)	1284.5 ± 141.1
Average relative propulsive force (%)	176.4 ± 14.5
Peak propulsive force (N)	1604.8 ± 233.4
Peak relative propulsive force (%)	219.6 ± 19.1
Peak landing force (N)	2968.3 ± 841.8
Average landing force (N)	803.3 ± 211.5
Relative peak landing force (%)	408.6 ± 109.9

***Velocity metrics***	

Average braking velocity (m/s)	−0.8 ± 0.1
Peak braking velocity (m/s)	−1.3 ± 0.2
Average propulsive velocity (m/s)	1.4 ± 0.1
Takeoff velocity (m/s)	2.4 ± 0.2
Peak velocity (m/s)	2.5 ± 0.2
Reactive strength index (RSI)	0.6 ± 0.1
Modified-RSI (mRSI)	0.3 ± 0.1

***Power metrics***	

Jump height (cm)	29.5 ± 5.8
Flight time (seconds)	0.5 ± 0.0
Jump momentum (kg ∗ m/s)	176.8 ± 14.6
Countermovement depth (cm)	−34.5 ± 5.8
Average braking power (watts)	−968.1 ± 208.7
Average relative braking power (W/kg)	−12.9 ± 2.1
Peak braking power (watts)	−1336.6 ± 312.6
Peak relative braking power (W/kg)	−17.9 ± 3.4
Average propulsive power (watts)	1670.1 ± 219.4
Average relative propulsive power (W/kg)	22.6 ± 3.3
Peak relative propulsive power (W/kg)	43.8 ± 6.9
Peak propulsive power (watts)	3228.4 ± 354.7
Time to stabilization (seconds)	1188.3 ± 161.5
Landing stiffness (N/m)	−3863.1 ± 2917.4
Stiffness (N/m)	−4647.5 ± 1286.5

*Note:* The preassessment is baseline data for all force, velocity, and power measures from the dual force plate, providing a reference point. The preassessment was not utilized for any correlations. Data are presented as the M ± SD. Units of measurement are abbreviated in the table as follows: % = percent.

Abbreviations: cm = centimeters, kg ∗ m/s = kilograms meters per second, m/s = meters per second, N = newtons, N/m = newtons per meter, N/s = newtons per second, s = seconds, W = watts, W/kg = watts per kilogram.

**Table 2:** The correlations between jump loads and force-based metrics focused on braking, propulsion, and landing forces.

	Correlation to duration (minutes)		Correlation to total jumps		Correlation to Jumps 38+		Correlation to Jumps 50+	
	Pearson's *r*	*p* value	Pearson's *r*	*p* value	Pearson's *r*	*p* value	Pearson's *r*	*p* value
Braking RFD (N/s)	−0.098	0.261	−0.021	0.814	−0.424	< 0.001	−0.478	< 0.001
Force at min displacement (N)	−0.079	0.363	−0.049	0.577	−0.453	< 0.001	−0.551	< 0.001
Relative force at min displacement (%)	−0.165	0.057	−0.131	0.131	−0.215	0.013	−0.194	0.025
Average braking force (N)	−0.073	0.401	0.040	0.647	−0.468	< 0.001	−0.573	< 0.001
Average relative braking force (%)	−0.143	0.100	0.013	0.884	−0.179	0.039	−0.147	0.090
Peak braking force (N)	−0.095	0.277	−0.043	0.619	−0.473	< 0.001	−0.570	< 0.001
Peak relative braking force (%)	−0.186	0.031	−0.122	0.159	−0.242	0.005	−0.220	0.011
Average propulsive force (N)	−0.020	0.816	−0.029	0.738	−0.389	< 0.001	−0.425	< 0.001
Average relative propulsive force (%)	−0.039	0.655	−0.077	0.375	−0.007	0.933	0.176	0.042
Peak propulsive force (N)	−0.062	0.478	−0.027	0.753	−0.433	< 0.001	−0.498	< 0.001
Peak relative propulsive force (%)	−0.125	0.150	−0.087	0.316	−0.067	0.441	0.067	0.439
Peak landing force (N)	0.053	0.540	0.146	0.092	−0.001	0.989	0.056	0.519
Average landing force (N)	0.050	0.566	0.074	0.397	−0.284	< 0.001	−0.372	< 0.001
Relative peak landing force (%)	0.051	0.557	0.156	0.072	0.213	0.013	0.363	< 0.001

*Note:* Data are presented as the correlation (Pearson's *r*) and significance level (*p* value) between daily jump loads (duration or jumps) and the force-based metrics from the CMJ tests. Units of measurement are abbreviated in the table as follows: % = percent.

Abbreviations: N = newtons, N/s = newtons per second.

**Table 3:** The correlations between jump loads and velocity-based metrics, as well as reactive strength index (RSI) and modified-RSI.

	Correlation to duration (minutes)		Correlation to total jumps		Correlation to Jumps 38+		Correlation to Jumps 50+	
	Pearson's *r*	*p* value	Pearson's *r*	*p* value	Pearson's *r*	*p* value	Pearson's *r*	*p* value
Average braking velocity (m/s)	0.142	0.102	0.092	0.288	−0.033	0.705	−0.130	0.134
Peak braking velocity (m/s)	0.120	0.169	0.030	0.734	−0.078	0.371	−0.187	0.030
Average propulsive velocity (m/s)	−0.113	0.193	−0.114	0.191	0.064	0.464	0.217	0.012
Takeoff velocity (m/s)	−0.074	0.395	−0.082	0.346	0.194	0.025	0.458	< 0.001
Peak velocity (m/s)	−0.078	0.373	−0.079	0.365	0.201	0.020	0.466	< 0.001
Reactive strength index (RSI)	−0.095	0.275	0.058	0.506	0.150	0.084	0.306	< 0.001
Modified-RSI (mRSI)	−0.097	0.267	−0.023	0.794	0.142	0.102	0.388	< 0.001

*Note:* Data are presented as the correlation (Pearson's *r*) and significance level (*p* value) between daily jump loads (duration or jumps) and the velocity-based metrics from the CMJ tests.

Abbreviation: m/s = meters per second.

**Table 4:** The correlations between jump loads and power-based metrics, jump height, jump momentum, countermovement depth, and stiffness.

	Correlation to duration (minutes)		Correlation to total jumps		Correlation to Jumps 38+		Correlation to Jumps 50+	
	Pearson's *r*	*p* value	Pearson's *r*	*p* value	Pearson's *r*	*p* value	Pearson's *r*	*p* value
Jump height (cm)	−0.067	0.441	−0.079	0.365	0.189	0.029	0.455	< 0.001
Flight time (seconds)	−0.049	0.570	−0.066	0.447	0.283	< 0.001	0.499	< 0.001
Jump momentum (kg ∗ m/s)	−0.064	0.461	−0.062	0.476	−0.278	0.001	−0.220	0.011
Countermovement depth (cm)	0.036	0.676	0.061	0.481	−0.224	0.009	−0.348	< 0.001
Average braking power (W)	0.132	0.127	0.005	0.950	0.343	< 0.001	0.380	< 0.001
Average relative braking power (W/kg)	0.150	0.083	0.042	0.628	0.043	0.625	−0.034	0.697
Peak braking power (W)	0.111	0.202	−0.039	0.655	0.268	0.002	0.270	0.002
Peak relative braking power (W/kg)	0.114	0.188	−0.005	0.957	−0.026	0.762	−0.135	0.121
Average propulsive power (watts)	−0.069	0.426	−0.088	0.309	−0.278	0.001	−0.207	0.016
Average relative propulsive power (W/kg)	−0.076	0.381	−0.106	0.224	0.069	0.428	0.282	< 0.001
Peak relative propulsive power (W/kg)	−0.072	0.411	−0.060	0.493	0.192	0.026	0.485	< 0.001
Peak propulsive power (W)	−0.085	0.327	−0.063	0.470	−0.154	0.076	0.036	0.676
Time to stabilization (seconds)	−0.030	0.740	−0.126	0.166	0.025	0.788	−0.005	0.958
Landing stiffness (N/m)	0.057	0.510	0.013	0.882	0.181	0.037	0.175	0.044
Stiffness (N/m)	0.013	0.882	−0.006	0.946	0.451	< 0.001	0.561	< 0.001

*Note:* Data are presented as the correlation (Pearson's *r*) and significance level (*p* value) between daily jump loads (duration or jumps) and the power-based metrics from the CMJ tests.

Abbreviations: cm = centimeters, kg ∗ m/s = kilograms meters per second, N = newtons, N/m = newtons per meter, N/s = newtons per second, s = seconds, W = watts, W/kg = watts per kilogram.

**Table 5:** The results of fisher's exact test, illustrating the probability of observing significant correlations between various jump variables (duration, total jumps, Jumps 38+, and Jumps 50+) and force plate metrics, emphasizing the stronger impact of high-intensity jumps on neuromuscular performance.

Jump variable	Correlated to force plate metrics	
	Significant correlations	Nonsignificant correlations
Duration	1	35
Total jumps	0	36
Jumps 38+	23	13
Jumps 50+	29	7

*Note:* The table displays the number of significant and nonsignificant correlations derived from the duration, daily jump loads, and subsequent force plate metrics.

## Data Availability

The data supporting the findings of this study are not publicly available as they were collected solely for the purposes of this research and contain information that could compromise participant privacy. However, anonymized data can be made available upon reasonable request to the corresponding author, subject to ethical and privacy considerations.
